# Vitamin D deficiency and polycystic ovary syndrome: an opinion and positioning article

**DOI:** 10.3389/fnut.2025.1602472

**Published:** 2025-07-09

**Authors:** Gao Song

**Affiliations:** ^1^Department of Kinesiology, College of Physical Education and Health Sciences, Zhejiang Normal University, Jinhua, China; ^2^Jinhua Wucheng Qiyuan Traditional Chinese Medicine Clinic, Jinhua, China; ^3^Jinhua Deren Rehabilitation Equipment Corporation, Jinhua, China

**Keywords:** vitamin D, polycystic ovary syndrome, insulin resistance, treatment, prevention

## 1 Introduction

Vitamin D is a steroid hormone whose primary function is to regulate bone growth and development, as well as calcium and phosphorus metabolism. Beyond its role within the skeletal system, vitamin D has been demonstrated to play a significant role in a number of non-skeletal diseases, including autoimmune diseases, diabetes, hypertension, inflammation and tumors (1–3). A substantial body of research has demonstrated the capacity of vitamin D to influence female reproductive functions, encompassing ovarian endocrine function, follicle formation, ovulation, and pregnancy. Polycystic ovary syndrome (PCOS) is one of the most prevalent endocrine disorders affecting women of childbearing age, with an incidence of up to 10% (4). The condition is characterized by ovulatory dysfunction and hyperandrogenism, which severely impair female reproductive function. Moreover, these patients frequently exhibit an array of systemic conditions, encompassing aberrant blood lipid and glucose metabolism, cardiovascular maladies such as hypertension (5). The etiology of PCOS remains multifactorial, involving a complex interplay of genetic and environmental influences, and existing research has not yet fully elucidated its pathogenesis (6). The potential for the vitamin D pathway to regulate PCOS-related symptoms, including ovulatory dysfunction, endocrine changes and insulin resistance, is a promising avenue for further research.

## 2 Vitamin D deficiency and ovarian reserve in patients with PCOS

PCOS is frequently characterized by irregular menstruation, infrequent ovulation or anovulation, and infertility, resulting in reproductive dysfunction in women of childbearing age. Researches have indicated that vitamin D has a significant role in female reproductive function. Anti-Müllerian hormone (AMH) is a reliable marker of ovarian reserve, produced by granulosa cells in primary, preantral and small antral follicles (7). Serum 25(OH)D levels have been observed to be positively correlated with AMH. In addition, it has been hypothesized that vitamin D supplementation may be effective in mitigating seasonal fluctuations in serum AMH (8). A prospective cross-sectional study found that vitamin D supplementation can maintain dominant follicles and improve ovarian reserve function (9). Consequently, assessment of vitamin D status can be used as an important adjunctive investigation in patients with PCOS.

## 3 Vitamin D deficiency and metabolic disorders in patients with PCOS

Researches have indicated a correlation between vitamin D deficiency and metabolic disorders in patients with PCOS (10). The expression of vitamin D receptor (VDR) in islets is known to be regulated by glucose, yet VDR expression is observed to be decreased in the diabetic mouse model. However, the study of transgenic mice overexpressing VDR in islet B cells has yielded promising results, with these mice demonstrating a capacity to prevent the onset of diabetes (11). A cross-sectional study found that serum vitamin D levels were significantly decreased in patients with PCOS, which was associated with higher insulin resistance and an unfavorable lipid profile (12). In addition, numerous clinical trials have demonstrated that vitamin D supplementation can reduce metabolic parameters, including blood lipids and cholesterol, and insulin resistance, as measured by a steady-state model (13–15).

Vitamin D has been demonstrated to influence glucose and lipid metabolism through multiple mechanisms. In peripheral insulin target cells, vitamin D has been observed to increase the expression of the insulin receptor, thus activating the glucose transporter. Vitamin D activates the transcription factor peroxide-proliferator activator receptor (PPAR) to increase insulin sensitivity. The role of the PPAR in the regulation of fatty acid metabolism in skeletal muscle and adipose tissue has been well documented (16). Conversely, vitamin D deficiency has been demonstrated to increase parathyroid hormone concentration, inhibit insulin secretion by islet B cells, and induce insulin resistance by regulating intracellular free calcium concentration (17).

## 4 Vitamin D deficiency and cardiovascular diseases in patients with PCOS

Patients diagnosed with PCOS frequently exhibit metabolic syndrome, characterized by obesity, abnormal blood glucose levels and dyslipidaemia, which collectively elevate the risk of developing cardiovascular disease. Vitamin D deficiency has been demonstrated to be associated with an increased risk of cardiovascular disease (18). The transverse section of the coronary artery in the PCOS rat model reveals fat infiltration, multiple inflammatory cells and focal calcified atherosclerotic plaques. In contrast, the coronary artery wall in the vitamin D treatment group exhibited normal characteristics, devoid of fat cells, plasma cells, and a minimal presence of inflammatory cells, thereby substantiating the cardioprotective efficacy of vitamin D (19). The potential mechanisms by which vitamin D exerts its protective effects on the cardiovascular system may be outlined as follows: firstly, vitamin D has the capacity to inhibit inflammation which is a fundamental pathogenesis of atherosclerosis; secondly, vitamin D can resist the hypertrophy of myocardial cells, which forms the basis of preventing congestive heart failure. Vitamin D regulates blood pressure by acting on endothelial cells and smooth muscle cells. Vitamin D deficiency has been associated with the renin-angiotensin-aldosterone system activation and contributes to the development of hypertension (20).

## 5 Effect of vitamin D supplementation on patients with PCOS

A prospective randomized controlled study found that the total testosterone, parathyroid hormone, free androgen index and hirsutism score of patients with PCOS were significantly decreased, while serum 25(OH)D, sex hormone binding globulin and phosphorus levels were significantly increased after 12 weeks of treatment with vitamin D. Furthermore, a substantial change was observed in the ovarian volume, follicle number, and regularity of the menstrual cycle (21). Karadag et al. (22) demonstrated that vitamin D supplementation can enhance the insulin sensitivity of patients with PCOS and reduce androgen levels, though it had no such effects on non-PCOS patients. In a study involving 67 patients with PCOS who were deficient in vitamin D (with 25(OH)D levels below 20 ng/ml) and 54 non-PCOS participants with vitamin D deficiency, a randomized controlled trial was conducted. The participants were administered 50,000 IU/week of cholecalciferol orally for 8 weeks and 1,500 IU/day of cholecalciferol orally for 4 weeks. Following the administration of vitamin D, a significant decrease in serum androstenedione levels was observed in the PCOS group (*P* = 0.007), accompanied by a substantial increase in the insulin sensitivity index (*P* = 0.001). A comprehensive review of the literature was conducted to ascertain the impact of vitamin D supplementation on patients with PCOS. Nine studies were identified that addressed this subject. In six of these studies, vitamin D supplementation led to a significant reduction in fasting blood glucose levels, an improvement in insulin resistance, and a decrease in serum fasting insulin. Four studies reported a decrease in serum triacylglycerol. In comparison with low-dose vitamin D (1,000 IU/day) and placebo, high-dose vitamin D (4,000 IU/day) has been shown to have a beneficial effect on hyperandrogenism. Furthermore, it has been demonstrated that high-dose vitamin D supplementation for a minimum period of 12 weeks can regulate the blood sugar level, insulin sensitivity, hyperlipidemia and hormone function of women with PCOS (23). These findings collectively indicated that vitamin D deficiency may have a role in the multifaceted pathogenesis of PCOS.

## 6 Conclusion

Vitamin D deficiency is a prevalent condition among individuals diagnosed with PCOS. This deficiency has been linked to a number of health complications, including follicular development disorder, metabolic disorder, cardiovascular disease and mental health issues. The supplementation of vitamin D has been demonstrated to regulate insulin resistance, lipid metabolism, and hormone levels in individuals diagnosed with PCOS. However, further research is required to elucidate the precise mechanism by which vitamin D exerts its effects on PCOS. In the diagnosis and treatment of patients with PCOS, especially those with metabolic syndrome, clinicians must be attentive to the potential for vitamin D deficiency and consider supplementation as appropriate. Concomitantly, further clinical studies are required to establish the most efficacious treatment guidelines. The underlying mechanisms by which vitamin D deficiency contributes to the development of PCOS remain to be fully elucidated and require further investigation.
